# Women’s Perspective of Breast Self-examination

**Published:** 2016-09

**Authors:** Motahare Pilevarzadeh

**Affiliations:** Master of Nursing Education, Lecturer of Nursing Midwifery Faculty, Jiroft University of Medical Sciences, Jiroft, Iran

**Keywords:** Breast Cancer, Breast Self-examination, Women’s Prospective

## Abstract

Breast cancer is the most prevalent cancer among Iranian women. Early diagnosis of the disease is vitally important in successful treating of it and reducing its mortality and care-cost burden. In our country, the major causes of mortality and other unfavorable complications of the disease are due to late referring of women. Breast Self -Examination (BSE) is a low-cost, low-risk, self-performed screening, which, according to the evidence from the literature improves the prospects for women’s survival. A content analysis with a qualitative approach was conducted in depth on women through semi-structured (individual and group) interviews. Based on the result analysis, one them related to women’s prospect BSE: Including Fearful feeling, with 2 Sub-themes A: Change body image B: Uncertain future. Training courses on breast cancer and BSE screening program for women (vulnerable poor population) is necessary so as to change their beliefs and culture in order to promote this health behavior.

## INTRODUCTION

Breast cancer is a stressful experience that leads to emotional anxiety and destructs one’s performance in performing daily tasks. The world’s statistics indicate increased number of breast cancer infection and quicker spread of it in developing countries, which have had low rate of breast cancer before (1). Despite technical advancements in surgery, chemotherapy, and radiotherapy, the rate of mortality caused by breast cancer has not changes for 50 years (2). The problem of screening can be an important factor to save mankind from a terrible nightmare called cancer. The availability of these methods, along with its outstanding results in reducing the severity and grading of cancer when they were discovered, indicate the importance of these tests (3). The most effective method of increasing the rate of survival in breast cancer is early diagnosis and treatment. Breast self- examination (BSE), clinical breast examination and mammography are methods used for the early diagnosis of breast cancer (4). for case-f Breast examination performed by the person herself, or breast self-examination, is a screening for case- Breast examination performed by the person herself, or breast self-examination, is a method ending in the early stages of breast cancer (5). The failure to refer heath care centers in the early stages of this cancer is affected by certain factors that include the women’s low level of knowledge of the realities of breast cancer, lack and/or neglect to do so, (BSE) Breast, social poverty, how to a late onset of bothersome symptoms of awareness of the importance of the individual examination and apply, of breast cancer (such as skin ulceration) and careless examination by the physicians. Statistics indicate the broad success of breast screening tests such as breast self-examination (6, 7). In Iran due to the effect of social and cultural impact of various factors on BSE, and also lack of comprehensive and systematic program to teach this approach to women, this practice is not done enough (8). This phenomenological study is to give a detailed description of a group of Iranian women’s prospect of survivorship from breast self-examination.

## MATERIALS AND METHODS

The purpose of this phenomenological study was to explore experiences of Women screening tests such as breast self-examination. A phenomenological approach was used to explore the essence of the participants’ experience of this phenomenon. Semi-structured interviews were conducted to gain a deeper understanding of those experiences.

Interpretative Phenomenological Analysis served as the methodology for this study by providing a structure for gathering detailed descriptions on how women providing a structure for gathering detailed descriptions on how women experience screening tests such as breast self-examination and how they perceive this underlying belief of s experience. The notion that there are multiple realities and gives meaning to people’s lives are the scholars in qualitative research (9, 10). In this method, the researchers seeks to discover the meaning behind the words, text and content analysis and uses data analysis which is, in fact, is the process of data analysis (11, 12). Sampling, from among the women referring to Jiroft health centers, the ones who had done BSE needed criteria to enter our study were chosen.

### Data Collection Methods

To obtain the desired information, each participant was individually interviewed, face-to face, using a semi-structured interview. All participants gave verbal and written consent to be audio taped prior to the interview. The interview questions were developed by the researcher I answered all of the questions by discussing them with my dissertation chair to eliminate any bias as I have disclosed personal experiences related to the phenomenon. The interview questions were based on the participants’ prospect related to screen breast cancer.

The initial interviews were no more than one hour in length. Participants were provided the opportunity to review the questions before the scheduled interview session.

A face-to-face, follow-up interview, lasting approximately 30 minutes, was conducted with each participant purposes of verification and clarification. Preliminary findings were shared with the for participants during the follow-up interview to ensure agreement with themes and to give the participants the opportunity to correct any misrepresented ideas or interpretations. Follow-up interviews were not recorded. The Written informed consent was obtained from each participant informed consent was summarized verbally for each participant to ensure that she understood her involvement in this process. Prior to e ach interview, the participant was asked to personal characteristics questionnaire attached to the informed consent. The personal complete the characteristics questionnaire consisted of items pertaining to the participants’ age, marital status, educational level.

I explained that this study will help to increase awareness of self- examination breast and how affects women of jiroft city.

The participants were informed that if at any time they did not feel comfortable with answering a question, they were not obligated to do so. They were also informed that each participant would be given a pseudonym to protect her identity.

The interview protocol was reviewed at the end to make certain that all the information was covered.

Thus, after completing interviews were transcribed immediately transcribed. At this stage, the interview was reviewed several times to gain a general sense of the text. Data were read verbatim and the first level coding process with emphasis on implicit and explicit content was bone by highlighting and identifying the sentences and paragraphs of the units of analysis. For each analysis, a higher code was given and the codes were extracted. Then the codes based on differences and similarities were classified. Depending on the relationship between the subset, a great number of subclasses were organized in other classes, in coding process, the codes were repeatedly controlled by the research team and in cases of contradictions, and inconsistencies were resolved by discussion and dialogue. After classification, classes were put together in form of a significant conceptual model. And the relationships between the classes were identified and the source code appeared (13-15). To determine the validity of the data in the study, continuous evaluation and analysis of data, analysis of data at the time of collection, review of the codes extracted from the participants, the study, scrutinizing of the analyzed data by two of the researchers of the qualitative research, and an ongoing involvement with the data were used (16). The researchers also carefully documented the details of the research and explained the research processes from first to end and so that the evaluation can be done by an external observer (17).

## RESULTS

After the data analysis, one of them was obtained from the data. It was fearful feeling, with 2 Sub-themes A: Change body image B: Uncertain future.

### Fearful feeling

Non-delayers experienced far less ambiguity about the implications of their symptom discovery and expressed more concern that the change they found might represent a serious health threat.

The women participating in this study stated that educating people to stay away from known risk factors, and encouraging them to healthy habits are among the first steps towards the prevention and control. And breast cancer screening is the best strategy for reducing cancer mortality. Similar findings emerge from the breast cancer screening literature which suggests that attenders for screening have more faith in the health care system than non- attenders, perceiving positive, rather than negative, consequences as the likely outcome of screening.

### A: Change body image

Some informants have expressed their unhappiness and distress on loss of body image due to the surgical treatment with one breast being removed or the scar that remained on her chest.

The theme category of altered body image included loss of feminine characteristic, long-lasting psychological effect, and stigmatization. Meanwhile, our informants also held the same view, which they have revealed the negative feeling of body image due to the surgical treatment of the breast cancer. Although some literature have suggested that women undergoing breast cancer.

Although some literature have suggested that women undergoing breast conservation surgery have better outcomes on selected psychosocial and quality of life measures then those of mastectomy.

### B: Uncertain future

We found that women with a history of breast cancer in the family had a higher degree of susceptibility regarding breast cancer in our study. Some of the informants have expressed their worries towards uncertain future with their loss of control over life and fear of recurrence (Table 1).

**Table 1:** Contents extracted from the data collected from participants in the study

Them	Sub-them

Fearful feeling	Change body image
	Uncertain future

## DISCUSSION

This study is important as it defines the breast cancer-related behaviors and beliefs of women.

The present study was conducted by the aim Women’s prospect of Breast Self-examination the. The results of the study showed that BSE to prevent breast cancer in addition to awareness dimension has psycho-mental dimension which is influenced by the social context and experience of each individual and is special to him. Since breast cancer is as a health threat and one of the major causes of death in women, it has caused great concerns and it is needed to raise people’s attitude (18). In general, attitudes make the performance possible (19). Cultural and attitudes about breast health and cancer concluded that many women are afraid of rumors and being abandoned by their spouse, his friends and family members do not pay much attention to BSE and had moderate attitudes towards BSE (20).

An attempt should be made to develop programs that are related heightening breast cancer knowledge, attitudes and practice of BSE (21). In a study by Ceber in Turkey lower levels of people’s attitude have been reported (22).

Education as an important factor in changing the attitude and approaches toward health and sanitation behavior. Culture and attitude can have an influence on health behavior such as BSE (1, 23). The socio-demographic characteristics of individuals can directly influence their attitude and indirectly affect health-related behavior (24). Women who were university graduates had higher health motivation, benefits related to BSE and self-efficacy than other woman (25). In the other study high level educated female physicians, according to the nurses, had higher health motivation, perceived BSE benefit, with the Woman’ health beliefs about mammography concludes that those woman who are university graduates have higher levels of health motivation and benefit Other studies the relationship between the women’s educational status and BSE performance (26).

Laimian stated that as the mission of health education guidelines are to warn against health threats, the requisite for effective training in controlling and preventing of breast cancer is attention to the structure of attitudes of women and proposed approach to encourage health monitoring (27).

Parsa (2008) recommends designing educational interventions based on famous psychological theories for breast cancer screening in developing countries (28) and Tavafian 2009 asserts on training programs related to BSE with the purpose of promoting self-effectiveness and considering the perceived barriers (29).

With careful attention to the importance of early preventive interventions for detection of diseases in which there is a special place for self-care and the high incidence and prevalence rates of breast cancer in the human population, experiences and researches, and emphasis on new scientific resources to promote the conduct of Obstetrics and Gynecology, (BSE) among women, especially women at risk for breast cancer is an undeniable necessity. And using this type of intervention, given that the costs involved in comparison to the benefits are affordable, can be given priority in the health care system.

Several studies suggest that improving the public’s attitude towards women screening for breast cancer can have a positive effect on behavior (30).

## CONCLUSIONS

It can be concluded that upgrading and modifying women’s health attitude, followed by creation of health behavior can guarantee a family’s health.
